# Flexibility Effects of a Flapping Mechanism Inspired by Insect Musculoskeletal System on Flight Performance

**DOI:** 10.3389/fbioe.2021.612183

**Published:** 2021-04-22

**Authors:** Sakito Koizumi, Toshiyuki Nakata, Hao Liu

**Affiliations:** ^1^Graduate School of Science and Engineering, Chiba University, Chiba, Japan; ^2^Graduate School of Engineering, Chiba University, Chiba, Japan

**Keywords:** Biomimetics, flapping robot, flexibility, insect musculoskeletal system, MAV (Micro Air Vehicle), robustness

## Abstract

Flying animals such as insects display great flight performances with high stability and maneuverability even under unpredictable disturbances in natural and man-made environments. Unlike man-made mechanical systems like a drone, insects can achieve various flapping motions through their flexible musculoskeletal systems. However, it remains poorly understood whether flexibility affects flight performances or not. Here, we conducted an experimental study on the effects of the flexibility associated with the flapping mechanisms on aerodynamic performance with a flexible flapping mechanism (FFM) inspired by the flexible musculoskeletal system of insects. Based on wing kinematic and force measurements, we found an appropriate combination of the flexible components could improve the aerodynamic efficiency by increasing the wingbeat amplitude. Results of the wind tunnel experiments suggested that, through some passive adjustment of the wing kinematics in concert with the flexible mechanism, the disturbance-induced effects could be suppressed. Therefore, the flight stability under the disturbances is improved. While the FFM with the most rigid spring was least efficient in the static experiments, the model was most robust against the wind within the range of the study. Our results, particularly regarding the trade-off between the efficiency and the robustness, point out the importance of the passive response of the flapping mechanisms, which may provide a functional biomimetic design for the flapping micro air vehicles (MAVs) capable of achieving high efficiency and stability.

## Introduction

Unmanned aerial vehicles (UAV), also known as drones, are widely used for various missions such as filming, surveillance, and transportation (Floreano and Wood, 2015; Liu et al., 2016). Because of the high-speed rotation of the thin propellers and their risk, the stability of their flight is crucial, especially when they fly in urban areas. However, it is still challenging for UAVs to fly safely because of the multi-scale unsteady aerial disturbances in the atmospheric boundary layer (Watkins et al., 2006). The sophisticated aeronautical theory for the aircraft cannot be applied directly to the UAVs. The aerodynamics at the low-Reynolds number regime is fundamentally different from the large-scale aircraft.

Animals such as insects and birds inhabit where drones are expected to operate. Their flight apparatus is thought to adapt to the flight in the unpredictable aerial environment through natural selection. Therefore, the bio-inspired strategy, such as flapping-wings, can greatly enhance the stability and reliability of conventional UAVs. For example, computational studies suggested that the unsteady separated flows on the flapping-wings can reduce the force fluctuation caused by the relatively large-scale turbulent flows (Engels et al., 2016; Ravi et al., 2016). The flapping-wings are, therefore, beneficial to enhance the stability of the UAVs.

**FIGURE 1:**
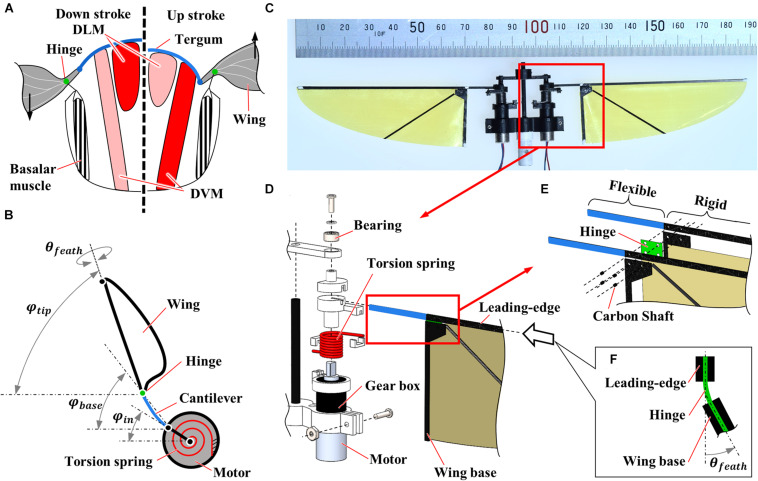
Flexible flapping mechanisms inspired by insect musculoskeletal system. **(A)** Schematic diagram of the musculoskeletal system of insects. Left and right half-frontal sections indicate the actuation of the indirect muscles for downstroke and upstroke, respectively. DLM and DVM represent dorsal longitudinal muscle and dorsal-ventral muscle, respectively. **(B)** Schematic diagram of the flexible flapping mechanism (FFM). A torsion spring, a cantilever, and a hinge are used as flexible components to realize a similar function with an insect musculoskeletal system. φ_*in*_, φ_base_, φ_tip_ and θ_feath_ indicate the positional angles at the input, the wing base, and the wing-tip, and the feathering angle, respectively. **(C)** Photograph of the fabricated FFM. **(D–F)** Exploded view of the CAD drawing of the mechanism; **(D)** Drive mechanism, **(E)** wing root, and **(F)** flexible hinge. The thickness of the flexible and rigid parts at the leading-edge is 0.4 and 0.8 mm, respectively.

Insects flap their wings by transmitting the alternative muscle contraction at the flexible skeletons (Figure 1A; Dudley, 2000). Due to the flexibility in the thorax, the power to decelerate the flapping-wings can be stored in the form of elastic energy at the thorax and released to accelerate the flapping-wings at the forthcoming stroke. The recycling of inertial power reduces the power consumption greatly (Dickinson and Lighton, 1995). In general, insects utilize their musculoskeletal flexibilities for locomotion, such as flapping (Ando and Kanzaki, 2016) and jumping (Mo et al., 2020a,b). Various micro aerial vehicles with flexible flapping-wings and mechanisms have been developed by taking inspiration from the insects’ flexible thorax. They utilize the flexibility to efficiently amplify the wing motion from the actuators such as piezoelectric actuators (Ma et al., 2013; Ozaki and Hamaguchi, 2020), electromagnetic actuators (Roll et al., 2016; Zou et al., 2016), and DC motors (Hines et al., 2014; Tu et al., 2020).

In addition to the power economy, the flexible structures may enhance the insects’ stability by responding to aerial disturbances passively. Flexible wings can respond to the gust by adaptively changing their shapes to reduce the force changes due to the gust (Nakata et al., 2018), enhancing the efficiency (Young et al., 2009; Nakata and Liu, 2012; Nian et al., 2019). Such an adaptive response is important to the animals and robots since the delay in the responses, mainly due to the sensory latency, greatly affects flight stability (Elzinga et al., 2012). Furthermore, the frequent adjustment of the wing kinematics can be energetically demanding. The enhancement of the stability through the adaptive response can, therefore, enhance the efficiency further. However, the effectiveness of the flexible mechanisms in terms of the gust response is poorly investigated despite its importance.

In this study, the effect of the flexibility in the flapping mechanism on the gust response of a flyer is investigated experimentally. We have fabricated a robot with insect-inspired flexible flapping mechanisms, slightly modifying the existing mechanism (Hines et al., 2014). The aerodynamic performance is investigated by the static experiment on the electric balance. The gust response of the flexible mechanisms is further investigated through wind tunnel experiments. The effect of the flexibility is investigated by constraining the flexible response mechanically.

## Materials and Methods

### Design and Fabrication of Flexible Flapping Mechanism

We have fabricated a motor-driven flexible flapping mechanism (FFM; Supplementary Video) by slightly modifying the mechanism developed by Hines et al. (2014). As illustrated in Figure 1B, the FFM consists of three main components: a driving motor, a torsion spring, and a wing with a cantilever and a hinge. The 6 mm geared micro coreless motors (ZWPD006006-26, Shenzhen ZHAOWEI Machinery & Electronics Co., Ltd.) directly drive each wing. The flapping motion of the wings is achieved without additional transmission by changing the direction of the electronic signal into the motors. The torsion spring mounted in parallel to both output shaft (Figures 1C,D) is expected to resonate by storing and releasing the elastic energy in response to the wing inertia, which reduces the power consumption at the motor (Li et al., 2018). The stiffness of the spring is crucial to generate a large lift with reasonable power consumption. Therefore, we have tested several different spring stiffness and evaluated the performance in terms of the wing kinematics, the lift, and the efficiency of the flapping-wings. The list of the springs used in this study is shown in Table 1. The torsion spring connector, the wing holder, and the frames were fabricated by cutting a plate of ABS (acrylonitrile butadiene styrene) using a CNC cutting machine (MDX-540, Roland DG Corp.).

**TABLE 1:** Parameters of flexible components.

Parameters	Values	Units
**Torsion spring**		
Hard	10.2	Nmm/rad
Medium	9.3	Nmm/rad
Soft	7.4	Nmm/rad
**Cantilever**		
Width	1	mm
Thickness	0.4	mm
Length from the rotational axis	14.4	mm
**Hinge**		
Width	4	mm
Thickness	80	μm
Length	0.2	mm
Young’s modulus	3.8	GPa

The wing part also has the flexibility at the wing base to increase the wingbeat amplitude and to adjust the feathering angle passively. The wing shape is a quarter ellipse with the length of semi-major and semi-minor axes of 70 and 25 mm, respectively (Tanaka et al., 2013). While the shape was originally designed by inspiring a hummingbird wing, we selected the wing dimension since it is close to that of large insects such as hawkmoths (Willmott and Ellington, 1997). The frame at the leading-edge and the wing base were made by cutting a carbon fiber-reinforced polymer (CFRP) sheet. The wing frames sandwich the hinge film and the wing membrane, and the carbon shafts are inserted to fix the components (Figure 1E). The leading-edge consists of the basal flexible cantilever and the distal rigid leading-edge. The flexible cantilever with a length of 14.4 mm from the motor output shaft and a thickness of 0.4 mm amplifies the positional angle. The membrane is a 5 μm thin polyimide film (Kapton, DU PONT-TORAY co., Ltd.), and the hinge is 80 μm thin polyimide film (Fuji Xerox Co., Ltd.). Flexible hinge modifies the feathering angle (angle of attack) of the wing passively so that it enhances efficiency while reducing the mechanical complexity of the system (Figure 1F; Supplementary Video). Passive feathering has been employed for various flapping-wing micro air vehicles (Ma et al., 2013; Zou et al., 2016). The detailed parameters of flexible components are found in Table 1. We did not change the wing structure throughout the experiments, but the wing flexibility can also affect flight performances. The structures of insect wings are finely tuned by the arrangement of wing veins and microstructures (Wootton, 2020). A proper combination of the flexibilities in mechanisms and wings may improve the performances, but it is beyond the scope of this study.

The whole mass of FFM is 4.0 g without a power supply cable. Additional electric components like battery, sensor, and control board would be equipped for free flight experiment in future work.

### Constrained Model

**FIGURE 2:**
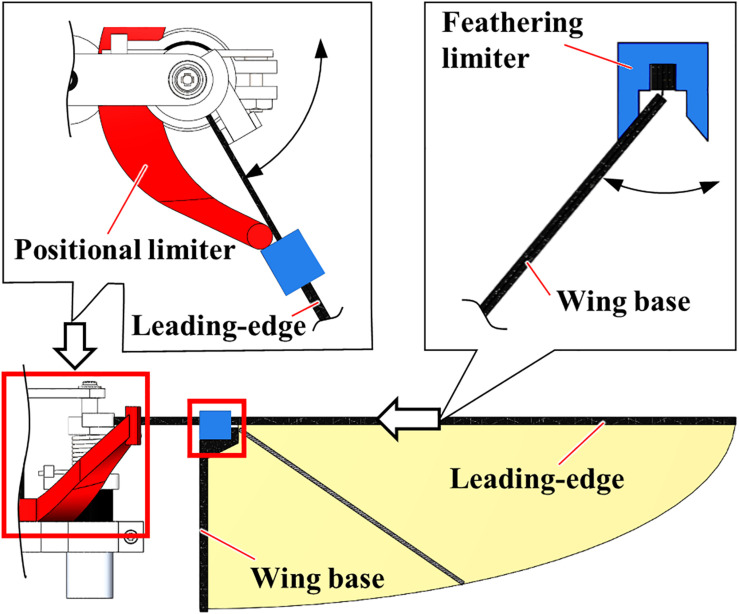
The “Constrained” model of FFM. The peak-to-peak positional (red) and feathering (blue) angles are constrained by additional components made of an ABS.

The “Constrained” FFM, shown in Figure 2, was fabricated to compare the effect of passive modification of wing kinematics on the robustness against the uniform frontal or lateral flow with the “Normal” FFM. Additional components constrain the maximum positional and feathering angles of the model. The positional limiter is located at the end of the cantilever to suppress the amplification of the amplitude, and the feathering limiter is mounted above the wing base.

### Experimental Setup

#### Kinematic and Force Measurements

In this study, we have evaluated the flight efficiency and robustness by fixing the FFM via force balance. Therefore, we did not include further complexity, such as body motions and active responses, in the evaluation. The “steady” setup is still useful because it simplifies the phenomenon to evaluate the performances separately from its flight dynamics.

**FIGURE 3:**
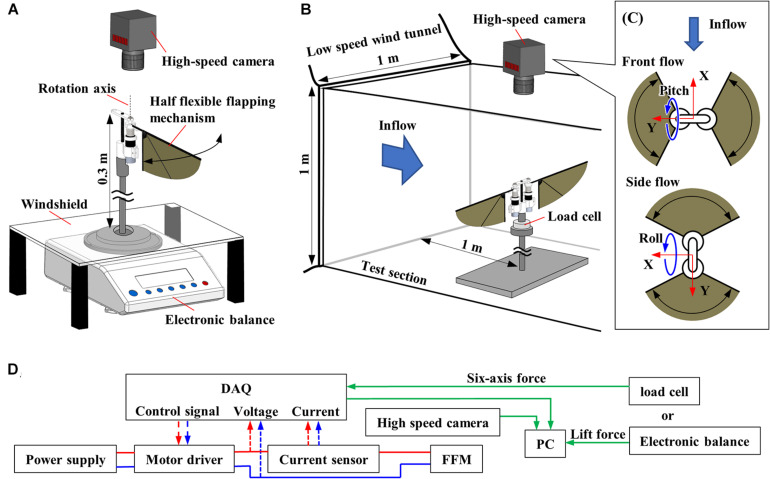
Experimental setup. **(A)** Schematic diagram of the setup for the measurement of the aerodynamic performance of FFM. **(B)** Schematic diagram of the wind tunnel experiment. **(C)** Definition of the coordinate system for the experiments with frontal or lateral flow viewed from the high-speed camera. **(D)** Diagram of data flow. The input voltage, input current, force, and wingbeat motion are all recorded with the DAQ.

Figure 3A shows the experimental setup of lift force measurement, which consists of an electronic balance (FZ-300I, A&D Co., Ltd., 1mN resolution), a windshield, and a high-speed camera (FASTCAM Mini AX, PHOTRON Ltd.). The half FFM was fixed on the top of the 300 mm steel bar, and the time-averaged lift force was measured by using the electronic balance below. The average lift force was sent to the PC through RS232C cable from electronic balance at 20 Hz, which was the maximum communication frequency of the device. A windshield covered a weighing pan to block the wake from the flapping-wing.

**FIGURE 4:**
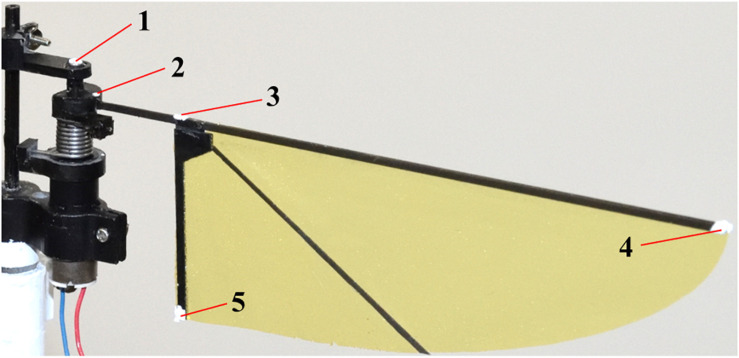
Five landmarks for the measurement of the wing kinematics.

A high-speed camera was placed above the system to film the wing kinematics at 2,000 fps. We tracked the five landmarks (Figure 4); rotation axis, fixed end of the cantilever, above the wing-base, wing-tip, and trailing edge. Recorded images were loaded into a program written for MATLAB (The MathWorks, Inc.), and the 2D coordinates of the landmarks in the images were acquired. The flapping angles are the positional angles at the input, the wing base and the wing-tip, and the feather angle (Figure 1B). The positional angles are the angular position of each point in the stroke plane. The positional angle at the input is the rotation angle of the motor output shaft. The positional angle at the wing base is the angle at the deformed cantilever from its mid-stroke position. The positional angle at the wing-tip is the rotation angle at the wing-tip from its mid-stroke position. The feathering angle is also called the geometric angle of attack and is the angle of the wing rotation around the spanwise axis on the wing pivot.

Figure 3D shows the diagram of the experimental setup. The FFM was driven with a PWM approximated sinusoidal voltage that was generated by a motor driver (SyRen 10A, Dimension Engineering LLC), which took control signal from a multifunction I/O device (USB-6343, National Instruments Corp.) and LabVIEW (National Instruments Corp.) program. Because the motor driver can change direction and rotation speed in proportion to the control signal between 0 and 5 V, the attitude of the robots can be controlled by changing the wing motion described in Ma et al. (2013). In addition to the lift force and the wing-tip trajectory, we also recorded input voltage and current supplied to the motor. Input current was measured by a Hall effect-based linear current sensor (ACS712-5A, Allegro MicroSystems, LLC). These data were recorded via the multifunction I/O device at 10 kHz.

In this study, three types of torsion spring and input voltage were tested to evaluate lift and efficiency at various wingbeat frequencies in the range of 12 to 25 Hz with an increment of 1 Hz. Each combination was measured three times, with a minute interval to cool the motor.

#### Wind Tunnel Experiment

We used a low-speed wind tunnel (Ikeda et al., 2018) to investigate how the passive modification of wing trajectory affects the robustness against the uniform frontal or lateral flow. As shown in Figure 3B, the FFM was placed on a six-axis force sensor (Nano 17 Ti, ATI Industrial Automation, Inc.) inside the test section. A high-speed camera was placed outside the test section above the FFM. Figure 3C indicates the view from the high-speed camera and the definition of wind direction and coordinates of the load cell. The force sensor was covered with 3D-printed parts (not illustrated) because the sensor was affected by lighting and wind. Six-axis force, input voltage, input current, and wingbeat trajectory were measured as described in the previous section. The torques at the load-cell were transformed to the torques around the center of gravity of FFM.

In this experiment, the input voltage was adjusted so that the lift-to-weight ratio is one without the wind. In order to confirm the reproducibility of the experiment, the measurement was conducted six times with each torsion spring stiffness. After each three-time measurement, the FFM was detached from the load-cell and attached again.

### Data Processing

The obtained data from previously described experiments were processed to calculate the efficiency. In this study, efficiency is defined as:

(1) $$\left\{ \begin{array}{c} \eta =\frac{P_{R\,F}}{P_{i\,n}}\\ P_{R\,F}=F_{z}\,w_{0}\\ w_{0}=\sqrt{\frac{F\,z}{2\,\rho \,A_{0}}}\\ A_{0}=\varphi \,R^{2}\,c\,o\,s\,\beta \end{array}\right.$$

where *P*_*RF*_ is the absolute minimum value of induced power, which is derived on the basis of the Rankine-Froude momentum (RFm) theory (Ellington, 1984), and *P*_*in*_ is input power. *F*_*z*_, *w*_0_, *A*_0_, φ, *R*, and β are mean lift, induced velocity, wingbeat area, amplified wingbeat amplitude, length of leading-edge, and stroke plane angle, respectively. The RFm efficiency is beneficial to calculate the ideal energetics of various combinations of frequency and input voltage. In this study, we assumed an ideal trajectory to calculate *P*_*RF*_. Therefore, φ and β were a constant value of π and 0, respectively.

## Results

### Aerodynamic Performance of FFM

**FIGURE 5:**
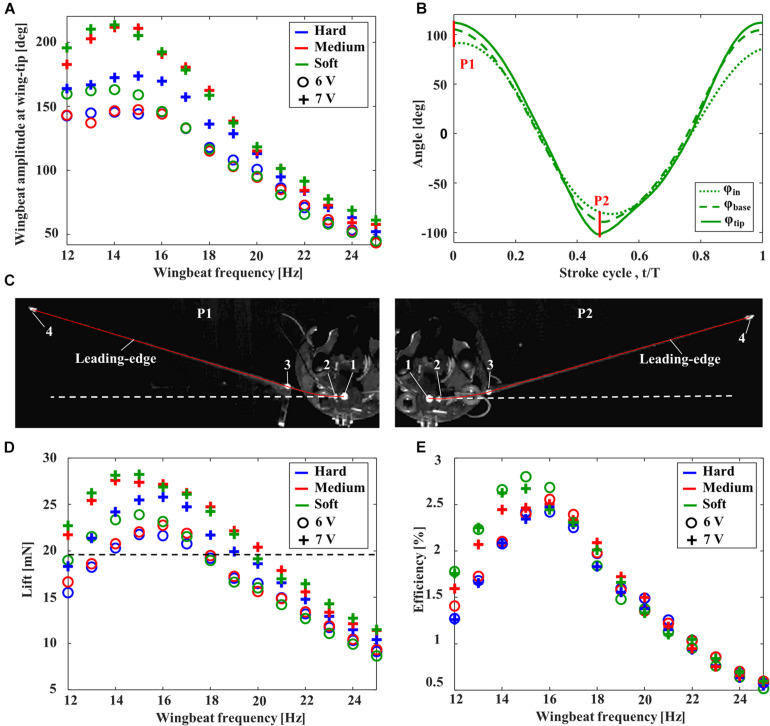
Aerodynamic performance of FFMs. **(A)** Effect of the wingbeat frequency on the wingbeat amplitude. **(B)** Time-series of the positional angles at the input (dotted line), wing base (broken line), and wing-tip (solid line) of the FFM (with “Soft” torsion spring) at 15 Hz with an input voltage of 7V. The red lines, labeled P1 and P2, correspond to the frames captured by the high-speed camera in **(C)**. **(C)** The snapshot at the stroke reversal. The solid red line and the white dashed line indicates the tracing of the leading-edge and the extended line of input angle (φ_*in*_), respectively. Effect of the wingbeat frequency on **(D)** the time-averaged lift and **(E)** the efficiency. The dashed line in **(D)** indicates the weight of half FFM.

The aerodynamic performance of FFM with various torsion spring stiffnesses and frequencies was evaluated in terms of the wing kinematics, the lift, and the efficiency. The wingbeat amplitude was increased by increasing the input voltage from 6 to 7 V, and was increased with decreasing wingbeat frequency from 25 Hz (Figure 5A). After reaching its maximum amplitude at around 14–15 Hz by the resonance, the amplitude was decreased gently or remained constant with decreasing wingbeat frequency. The frequency of maximum wingbeat amplitude was increased with increasing stiffness of torsion spring from “Soft” (14 Hz) to “Hard” (15 Hz).

The maximum wingbeat amplitude of each input voltage was achieved by “Soft” torsion spring at around 14 Hz. Figure 5B shows the time-series of each positional angle of the “Soft” torsion spring at 14 Hz with an input voltage of 7 V. While the peak-to-peak input amplitude was 172 degrees, the peak-to-peak amplitude at the wing-tip was increased up to 213 degrees by the deformation at the cantilever. The passive deformations were maximized at the stroke reversal, where the inertial force due to the wing acceleration is maximized (Figure 5C).

Unsurprisingly, the large lift was generated when the wingbeat amplitude was larger, as shown in Figure 5D. Therefore, the passive deformation in the spring and the cantilever can significantly affect the lift force generation. In this study, the maximum lift-to-weight ratio was 1.4, with the combination of “Soft” torsion spring and the wingbeat frequency at 15 Hz. Figure 5E clearly shows that higher efficiency can be achieved when the wingbeat amplitude and the lift are higher. In the case of a lift-to-weight ratio above 1, the more flexible torsion spring can achieve higher efficiency.

### Wind Tunnel Experiment

The FFM can generate lift force with the highest efficiency at 15 Hz with “Soft” torsion spring, while, with “Hard” and “Medium” torsion spring, the highest efficiency was achieved at 16 Hz (Figure 5E). Since the peak-to-peak positional amplitude of “Medium” and “Soft” torsion spring exceeded 180 degrees depending on the input voltage, the wingbeat frequency was set to 16 Hz to avoid wing interference during the wind tunnel experiment with full FFM.

**FIGURE 6:**
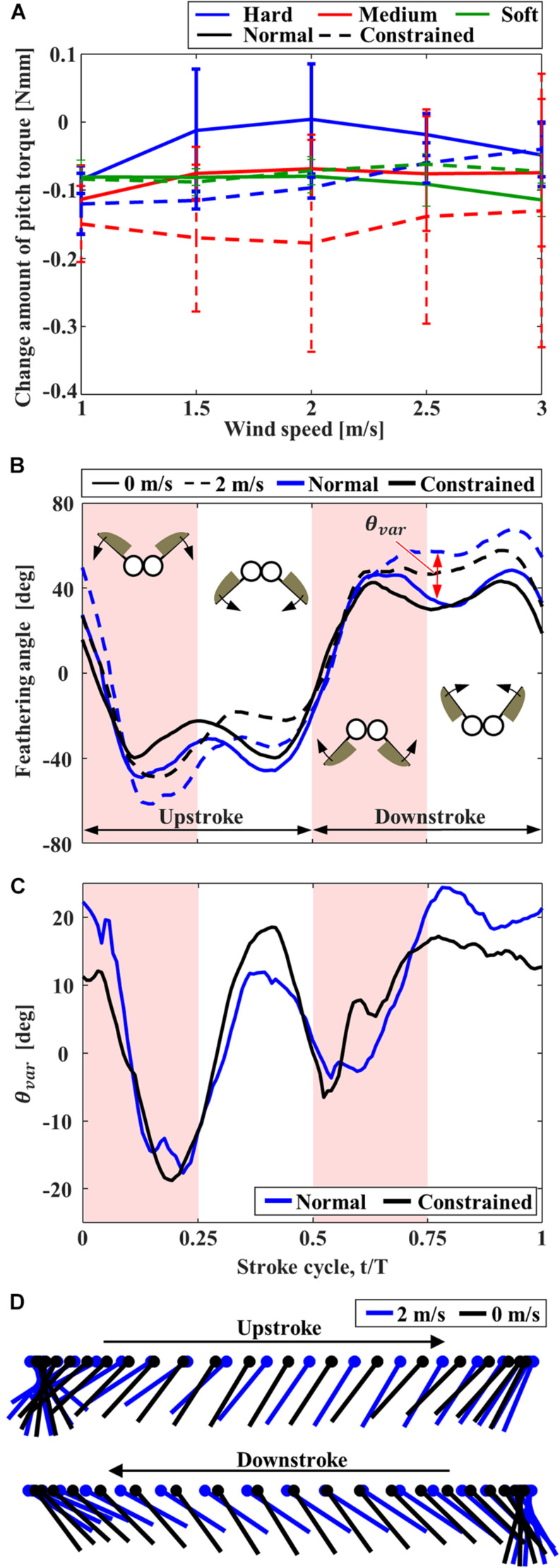
The effect of frontal wind on the pitching torque and feathering angle of FFM. **(A)** Effect of the frontal wind speed on the variation of pitching torque from those without wind. **(B)** Time-series of the feathering angles of “Normal” and “Constrained” FFM with “Hard” torsion spring plotted from the beginning of upstroke. **(C)** Increment of the feathering angle due to the frontal wind of 2 m/s. **(D)** Angular motion of the wing chord of “Normal” FFM with “Hard” spring without wind (black) and with the frontal wind of 2 m/s (blue).

The pitching torques due to the frontal wind were negative (Figure 6A), and therefore the FFM pitches up by the frontal wind. At the medium wind speed, the pitching torques on the “Normal” FFM were closer to zero than the “Constrained” FFMs, which is beneficial to enhance the stability. The pitching torque on the “Normal” FFM with the “Hard” spring was the smallest within the range of the study. The feathering angles of both “Normal” and “Constrained” FFMs with the “Hard” spring were increased between t/T = 0.1 – 0.3 by the frontal wind, but they were decreased between t/T = 0.3 – 0.5 (Figures 6B,D) by the frontal wind. By contrast, the feathering angles were increased during downstroke by the frontal wind. The variations of the feathering angle due to the frontal wind were similar during the upstroke, while the feathering angle of the “Normal” FFM was increased more than the “Constrained” mechanism during the upstroke (Figure 6C).

**FIGURE 7:**
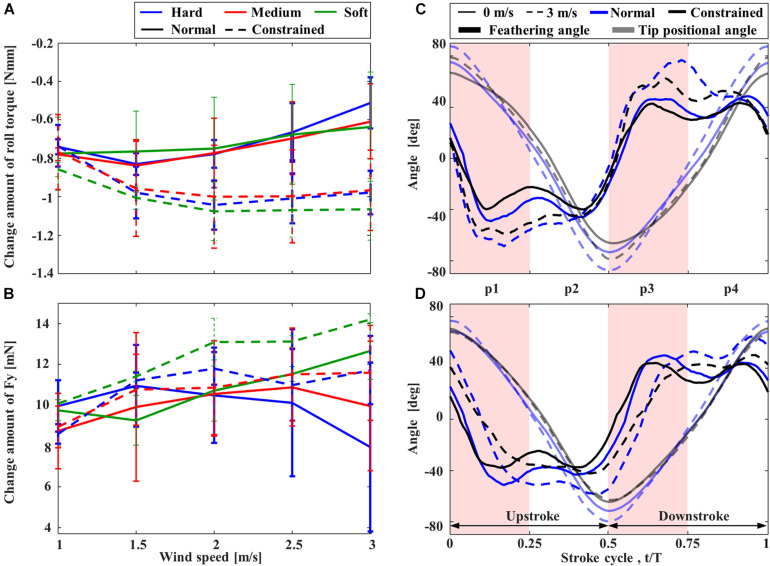
The effect of lateral wind on the rolling torque and feathering angle of FFM. Effect of the lateral wind speed on the variation of **(A)** rolling torque and **(B)** lateral force from those without wind. Time-courses of the feathering angles of the wings at **(C)** upstream and **(D)** downstream.

Rolling torques on the mechanisms induced by the lateral wind were also negative (Figure 7A), indicating that the rolling torques were applied in the direction of downstream flow compared to the condition without wind. Nevertheless, compared to the “Constrained” FFM, the “Normal” FFM suppressed the torque change, especially when the higher wind speed. Again, the torque on the “Normal” FFM with the “Hard” spring was the smallest within the range of the study, especially when the higher wind speed. The lateral force, *F*_*y*_, was also reduced in the “Normal” FFM compared to the “Constrained” FFM at higher wind speed (Figure 7B). Therefore, the effect of the lateral wind was reduced by the passive change in the wing kinematics. In the “Constrained” mechanisms, there was no remarkable difference in the rolling torque among the mechanisms with the different spring stiffness. The lateral wind increased the positional angles at the wing-tip and the feathering angles of both mechanisms with “Hard” spring at the upstream wing (Figure 7C). At the downstream wing (Figure 7D), the amplitudes of the feathering angles were relatively unchanged, but the phase was delayed by the lateral wind. The wing-tip positional angle was increased in the “Normal” FFM, but it was unchanged in the “Constrained” FFM. The feathering angle of the upstream wing of the “Normal” mechanism in the middle of the downstroke was increased (p3 in Figure 7C). The feathering angle of the downstream wing of the “Constrained” mechanism was relatively unchanged at the end of each half-stroke, while the feathering angle of the “Normal” mechanism was increased (p2 and p4 in Figure 7D).

## Discussion

### Effects of the Flexibility in the Flapping Mechanism on the Aerodynamic Performance

We have fabricated the flexible flapping mechanism (FFM) inspired by a flexible insect musculoskeletal system. The mechanism in this study was the modification from the original design of Hines et al. (2014). They suggested the importance of the wing offset from the rotation axis. In this study, we have replaced the offset part with the flexible cantilever, which can further increase the wingbeat amplitude by passive deformation. As shown in Figure 5C, the cantilever deforms at the stroke reversal and amplifies the wingbeat amplitude. This function of the flexible cantilever at the wing base is an essential feature for realizing efficient wing motion. Only one type of cantilever was tested in this study, but it is necessary to test the various designs to create a more efficient and robust FFM.

The flapping robots and animals can benefit energetically from the flexibility in the mechanisms. The FFM in this study is qualitatively similar to the insect thorax, having three different elastic elements: a torsion spring, a cantilever, and a hinge (Figures 1A,B). As a result of the aerodynamic performance measurements, it was found that the FFM can generate more lift force than its own weight by appropriately designing the flexible components. The efficiency can be enhanced further by selecting the stiffness of the elements and the wingbeat frequency. The stiffness of each element determines the resonance frequency at which the FFM should operate to enhance efficiency. The enhancement of the efficiency by the flexible components has already been confirmed in fruit flies (Dickinson and Lighton, 1995), and this robotic study further confirmed the importance of the flexibility at the components.

### Effect of the Flexibility in the Flapping Mechanism on the Robustness Against the Disturbance

The passive change of the wing kinematics in response to the aerial disturbances affects the stability of the flapping-wing flyer. In this study, the passive feathering reduced the forces and the torques induced by the frontal or lateral wind. A similar effect by the flexible wing has been suggested by a computational study (Nakata et al., 2018). While we need to test with more variation in the stiffness to understand the physical mechanisms behind the robustness, the flapping mechanism can reduce the effect of disturbances further by appropriately designing the stiffness. It should be noted that there is a possibility that the appropriate stiffness of the flexibilities could depend on both wing and body shapes. Previous studies have reported the importance of the wing shape and structure (Keennon et al., 2012; Nan et al., 2017) as well as the tail shape (Nan et al., 2020) to improve mechanical efficiency and aerodynamic lift force production. Therefore, it will be our future work to experimentally examine the effects of various wing shapes and flexibilities to generalize our results and improve FFM performance.

**FIGURE 8:**
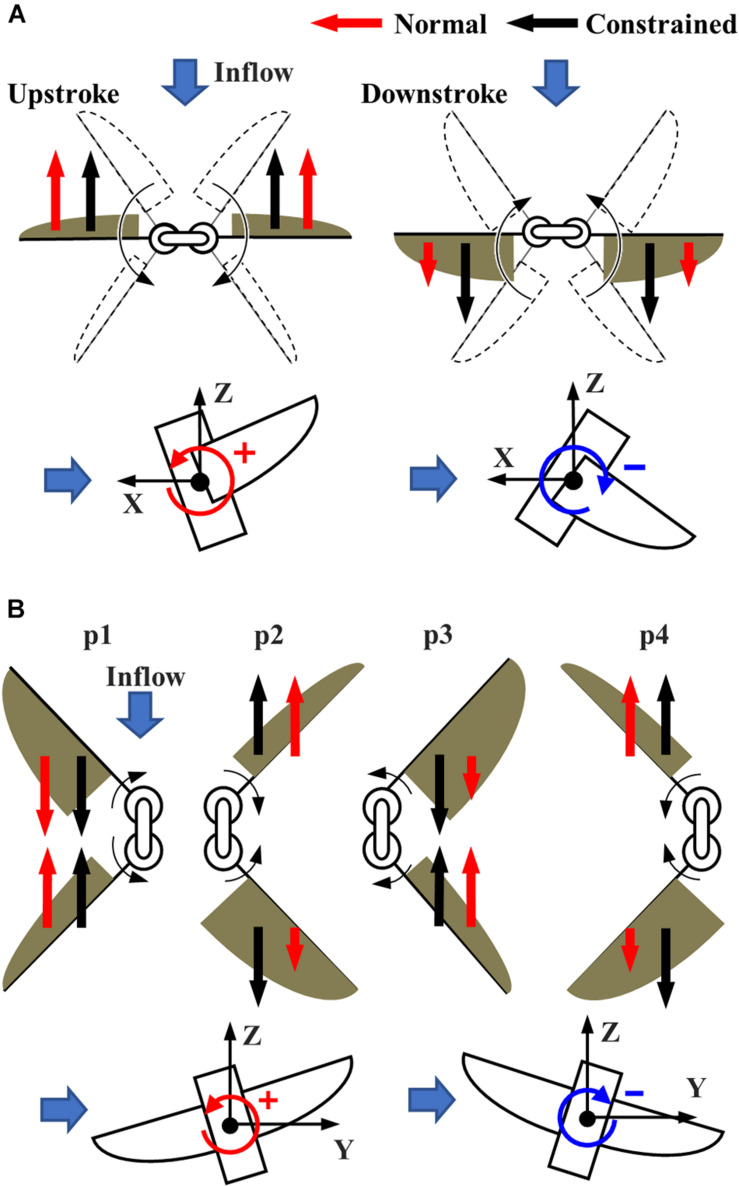
The torque reduction mechanism under the **(A)** frontal and **(B)** lateral wind. **(A)** The reduction of the drag during the early half of each half-stroke reduces the pitching torque under the frontal wind. **(B)** The reduction of the drag during early half of each half-stroke at the upstream wing (p3) and the latter half of each half-stroke at the downstream wing (p2 and p4) reduce the rolling torque under the lateral wind.

The pitching torque due to the frontal gust can be reduced by the passive adjustment of the feathering angle. The increase in the feathering angle during downstroke (Figure 6C) reduces the angle of attack of the wing, which reduces the drag on the wing greatly (Figure 8A). It should be noted that the feathering angles during downstroke are relatively more important than those during the upstroke because the relative speed of the wing is increased during downstroke by the frontal wind. The drag reduction will result in the pitching-up torques since the stroke plane is above the center of mass (Liang and Sun, 2013).

The passive adjustment of feathering can respond to the lateral wind similarly to the frontal wind. The relative increase in feathering angles, or the reduction in angle of attack, reduces the drag on the upstream wing during the early half of each half-stroke (especially p3 in Figure 7C). Similarly, the passive reaction reduces the drag on the downstream wing during the latter half of each half-stroke (p2 and p4 in Figure 7D). The reduction of the drag at the appropriate timing in the “Normal” FFM relatively increases the lateral force (Figure 8B). Therefore, it increases the positive rolling torque at the timing, which results in the reduction of the absolute rolling torques (Figure 7A). In other words, the model that can passively change the wing kinematics in response to lateral disturbances can reduce the rolling torques acting on the FFM compared to a model with constrained wing motion.

There is a trade-off between efficiency and robustness against the disturbances. While the FFM with the “Hard” spring was most robust against the frontal and lateral winds (Figures 6A, 7A), the efficiency of the model was not as high as FFM with the “Soft” spring (Figure 5E). The trade-off is crucial when designing the UAV. Even if the efficiency is high in the quiescent air, the soft mechanism may require more power to stabilize the attitude under unpredictable disturbances by frequently adjusting the flapping-wing kinematics. Furthermore, flexible structures may be harder to control in general, as we observe for flexible robots (Lee et al., 2017). Therefore, the FFM structure should be carefully designed with the consideration of the aerial environment where the UAV will operate.

## Data Availability Statement

The raw data supporting the conclusions of this article will be made available by the authors, without undue reservation.

## Author Contributions

TN conceived the study with SK and HL. SK, TN, and HL designed the experiments. SK performed the experiments. SK and TN analyzed the data. All authors contributed to the final version of the manuscript and approved the submission.

## Conflict of Interest

The authors declare that the research was conducted in the absence of any commercial or financial relationships that could be construed as a potential conflict of interest.
